# BRAIN GLUTATHIONE, MITOCHONDRIA, GLUCOSE METABOLISM, AND COGNITION IN AGED MICE: IMPACT OF GLYNAC SUPPLEMENTATION

**DOI:** 10.1093/geroni/igae098.0416

**Published:** 2024-12-31

**Authors:** Premranjan Kumar, Rajagopal Sekhar

**Affiliations:** Baylor College of Medicine, Houston, Texas, United States; Baylor College of Medicine, Houston, Texas, United States

## Abstract

Alzheimer’s disease (AD) remains unsolved, and is associated with brain defects including impaired glucose uptake, mitochondrial dysfunction, inflammation and glutathione deficiency. These defects also occur in age-related cognitive decline (ACD). Because mice do not naturally develop AD, mouse models of AD have been developed and studied. However, such mouse models are highly artificial because defects do not occur naturally and are artificially induced in mice. To understand the mechanistic basis of cognitive decline in aging, it is important to investigate these defects directly in the brain, which is only possible in animal models as performing brain biopsies in humans is not practical. To overcome these challenges, we studied glutathione deficiency, oxidative stress, glucose uptake, mitochondrial function, inflammation, genomic damage and neurotropic factors directly in the brains of old-mice (OM, 90-weeks)) and young-mice (YM, 20-weeks), and also measured cognition (maze-running). Compared to YM, OM had the following defects in the brain: (a) lower glutathione concentrations; (b) elevated oxidative stress (TBARS); (c) lower immunoblot expressions of: GLUT1 and GLUT3 (brain and neuronal glucose uptake); PGC1a; electron-chain complexes and PINK1 (mitochondria), TSPO (inflammation), pH2AX (genomic damage), and BDNF, GDNF and NGF (neurotrophic factors); (d) impaired mitochondrial respiration (high-resolution respirometry); (e) poor cognition (maze-running time). After 8-weeks of supplementation with GlyNAC (combination of glutathione precursors glycine and N-acetylcysteine), all of these defects improved/corrected in the brains of OM and cognitive performance improved significantly. The results of this study have important implications for improving brain health and cognition in human aging and in AD.

